# Mood, anxiety, and alcohol use disorders and later cause-specific sick leave in young adult employees

**DOI:** 10.1186/s12889-016-3427-9

**Published:** 2016-08-03

**Authors:** Fartein Ask Torvik, Ted Reichborn-Kjennerud, Line C. Gjerde, Gun Peggy Knudsen, Eivind Ystrom, Kristian Tambs, Espen Røysamb, Kristian Østby, Ragnhild Ørstavik

**Affiliations:** 1 Norwegian Institute of Public Health, Domain for Mental and Physical Health, P.O. Box 4404, Nydalen, 0403 Oslo Norway; 2 University of Oslo, Institute of Clinical Medicine, P.O. Box 1171, Blindern, 0318 Oslo Norway; 3 University of Oslo, Department of Psychology, Section of Health, Developmental and Personality Psychology, P.O. Box 1094, Blindern, 0317 Oslo Norway; 4 University of Oslo, School of Pharmacy, PharmacoEpidemiology and Drug Safety Research Group, P.O. Box 1094, Blindern, 0317 Oslo Norway

**Keywords:** Anxiety, Depression, Specific phobia, Sick leave, Sickness absence, Functional impairment, Occupational health

## Abstract

**Background:**

Mental disorders strongly influence work capability in young adults, but it is not clear which disorders that are most strongly associated with sick leave, and which diagnoses that are stated on the sick leave certificates. Better knowledge of the impairments associated with different mental disorders is needed for optimal planning of interventions and prioritization of health services. In the current study, we investigate the prospective associations between eight mood, anxiety, and alcohol use disorders, and later sick leave granted for mental, somatic, or any disorder.

**Methods:**

Lifetime mental disorders were assessed by structured diagnostic interviews in 2,178 young adults followed for eight years with registry data on sick leave. Relative risk ratios were estimated for the associations between each mental disorder and the different forms of sick leave.

**Results:**

All included diagnoses were associated with later sick leave. In adjusted analyses, major depressive disorder and generalized anxiety disorder were the strongest predictors of sick leave granted for mental disorders, whereas social anxiety disorder and specific phobia were the strongest predictors of sick leave granted for somatic disorders. Specific phobia and major depressive disorder had the highest attributable fractions for all-cause sick leave.

**Conclusions:**

Mood and anxiety disorders constituted independent risk factors for all cause sick leave, whereas alcohol use disorders seemed to be of less importance in young adulthood. Disorders characterised by distress were most strongly associated with sick leave granted for mental disorders, whereas disorders characterised by fear primarily predicted sick leave granted for somatic conditions. A large part of all sick leave is related to specific phobia, due to the high prevalence of this disorder. The impairment associated with this common disorder may be under-acknowledged, and it could decrease work capacity among individuals with somatic disorders. This disorder has good treatment response and may be overlooked as a target for interventions aimed at prevention of sick leave.

## Background

Individuals with mental disorders [1–6] or symptoms of mental disorders [7–10] have higher rates of all-cause sick leave. Sick leave is a strong indicator of impairment, leads to substantial economic losses [11], and a risk for permanent work exclusion [12, 13]. Mental disorders are particularly important for work impairment in young adulthood, as somatic diseases become increasingly important with age. Young adults also have most of their careers ahead of them, so poor workforce integration can affect them for many years, leading to high costs [14]. Furthermore, official sick leave statistics probably underestimate the true burden of mental disorders [5, 15], as mental disorders can be a risk factor for sick leave granted for somatic disorders. Despite its importance, the level of and “official reason” for sick leave associated with different common mental disorders is not known. Knowledge of the impairments associated with different mental disorders is needed in planning of interventions and prioritization of health services.

Studies with different methodology varyingly find anxiety or depression to be most predictive of sick leave: In a small study using diagnostic interviews, depression was more strongly associated with subsequent sick leave than was anxiety [4]. In another study with a much larger sample, self-reported symptoms of depression did not predict sick leave independently of anxiety [7]. Considering other measures of work impairment, studies with psychiatric diagnoses have varyingly found anxiety [16, 17] or mood disorders [18–20] to have the strongest influence on impairment. Smaller effects have been seen for alcohol and substance use disorders [17, 18, 20, 21]. Unfortunately, previous studies either use self-reported symptom measures instead of diagnoses [7, 8, 10], broader categories of mental disorders instead of specific diagnoses [1, 3–6, 9, 21], small samples [1, 6, 22], or other work-related impairment measures than sick leave [17–21]. The prospective associations of different mood, anxiety and alcohol use disorders with later sick leave therefore remain largely unexplored.

A few studies have shown that mental disorders or symptoms of mental disorders do not only predict sick leave granted for mental, but also somatic disorders [1, 3, 5, 6]. Nevertheless, most studies did not distinguish between diagnoses on the sick leave certificates. It is therefore also unknown to what degree diagnostically assessed mental disorders predict sick leave explicitly granted for mental disorders and to what degree the different mental disorders are risk factors for sick leave granted for somatic illness.

The present study adds to previous knowledge by using diagnostic interview data and by being able to distinguish between different sick leave diagnoses in high-quality registries. We use the linkage between diagnostic interviews and population-based registries in order to investigate the associations between eight common lifetime Diagnostic and Statistical Manual of Mental Disorders (DSM)-IV mental disorders covering mood, anxiety, and alcohol abuse disorders, and later sick leave granted for mental disorders, somatic disorders, or any disorder in young adult employees.

## Methods

### Sample and assessment

The sample for the current study originated from the Norwegian Twin Registry. Twins were identified through the Medical Birth Registry of Norway, established on January 1, 1967. All twins born between 1967 and 1979 who were alive and had a known address in 1998 were invited to complete a questionnaire. Out of 12,700 invited individuals, 8,045 (63.3 %) responded after one reminder. Diagnostic interviews were then conducted between 1999 and 2004 among a subsample of complete twin pairs who had responded to the questionnaire and had given consent to be contacted again later. Due to technical problems, 68 twin pairs were drawn directly from Norwegian Twin Registry. The response rate was 43.5 % (2,801 out of 6,442). Sex predicted participation in the initial questionnaire, whereas only older age and monozygosity predicted attrition from the questionnaire to the interview study, out of 45 potential predictors [23]. Permission to link data to official registries was granted for 2,770. We only included individuals who were employed at least 50 % of the days in the year they were interviewed (78.7 % of men and 78.4 % of women). This criterion was needed to ensure that mental disorders were assessed among employed young adults. In the final sample of 2,178 individuals, 856 (39.3 %) were men, and age at interview (baseline) spanned from 19 to 36 years, with a mean of 28.6 (SD = 3.8). The subjects were followed from the year of the interview until the end of 2008 (average follow-up time 8.0 years; SD = 1.3).

### Measures

#### Lifetime mental disorders

Lifetime mental disorders were assessed using a Norwegian version of the computerized Munich Composite of International Diagnostic Interview [24]. This is a structured diagnostic interview developed by the World Health Organization (WHO) for the assessment of the DSM-IV and the International Classification of Diseases (ICD)-10 lifetime diagnoses. The interview has previously shown good test–retest and interrater reliability [25, 26]. The interviewers were mostly psychology students in their final part of training or experienced psychiatric nurses. They received a standardized training program by teachers certified by the WHO. Most of the interviews were conducted face to face, but for practical reasons 8.3 % were interviewed by phone. In the present study, we used dichotomous lifetime diagnoses of major depressive disorder, dysthymia, generalized anxiety disorder, social anxiety disorder (social phobia), specific phobia, panic disorder, agoraphobia, and alcohol use disorder.

#### Employment and sick leave

The Historical-Event Database is a governmental registry that contains data on economic activity for the entire population. Information regarding employment, taxation, and social security benefits, including sickness benefits exceeding 16 days, is recorded in the registry. Sick pay is covered by the mandatory Norwegian Insurance Scheme after 16 days. Due to the economic incentive, it is unlikely that employers should refrain from reporting these sick leaves. Sick leave periods shorter than 17 days were not included in this study. For each day from January 1, 2000 to December 31, 2008, we had information on whether participants were registered as employed, on sick leave, and if available, diagnoses. In this registry, diagnoses are coded according to the International Classification of Primary Care-2 (ICPC-2) [27]. We constructed three sick leave variables: Sick leave granted for mental disorders, sick leave granted for somatic disorders, and all-cause sick leave. Sick leaves granted for disorders in chapter W of ICPC-2 (“Pregnancy, childbearing, family planning”) accounted for 19.6 % of sick leave days, and were excluded from the present analyses. Pregnancy related sick leave is restricted to a subgroup of the sample and is likely to have causes specific to pregnancy. Sick leave granted for mental disorders included sick leaves with diagnoses in the P-chapter of ICPC-2 (“Psychological”) and accounted for 20.5 % of sick days. Sick leave granted for somatic disorders included all other diagnostic chapters of the ICPC-2 and accounted for 53.0 % of sick days, of which the L-chapter (“Musculoskeletal”) was the most common (33.0 % of sick days). Sick leaves with unknown diagnosis (6.9 %) were included in the total, but not in the mental or somatic categories. The number of sick leave days was summed for each subject. All tax-reported employments are registered in the database, which made it possible to calculate the rate of employment. If individuals had several employments on the same date, it was counted as one work day. Public holidays and paid vacation of either 4 weeks and 1 day or 5 weeks per year is registered as workdays, but the rate of these is essentially the same for everyone, and therefore unlikely to affect the results. As the degree of employment varied between subjects, the sick leave variables were defined as a ratio (0–100 %) of days lost to sick leave to the number of potential working days.

#### Covariates

Information on sex, year of birth, and socioeconomic background was available from the registries. Socioeconomic background was defined as the parents’ education when the index person was 16 years old and was used as an ordinal variable with four levels (elementary school, high school, shorter tertiary education, and longer tertiary education; determined by the parent with the highest education). Preliminary analyses were run both controlling for and not controlling for parental education and showed that the inclusion of these variables did not noticeably affect the results.

#### Statistical analyses

Associations were investigated using multiple regression analyses. We used a binomial distribution in order to calculate the probability of sick leave occurring *k* out of *n* days. The assumption of a negative binomial distribution is suitable in the case of overdispersion. As there were only negligible differences in the results when using the binomial and the negative binomial distribution, we report results from binomial regression. A log link function was chosen to obtain results expressed as relative risks (RRs). In our case, the RR reflects the risk of having sick leave on a given workday. Thus, a RR of 2.00 indicates that the level of sick leave is twice as high among individuals with the risk factor. Generalized estimating equations were applied to account for statistical dependency between twins in a pair. We used robust estimation of standard errors.

We first assessed the associations between each of the eight diagnoses and all-cause sick leave, and then obtained adjusted estimates by entering all disorders into the regression models simultaneously. The analyses were repeated for sick leave granted for mental disorders and sick leave granted for somatic disorders. All analyses were adjusted for sex, age, and parental education.

Population attributable fractions (PAFs) indicate the proportion of sick leave preventable if a risk factor is removed from the population, assuming that the relationship is causal [28]. PAFs were calculated from the regression results using the average attributable fraction approach [29].

All analyses were run in R 3.1.2 [30] with the “gee” package [31].

## Results

Lifetime prevalences of the eight mental disorders among the employed participants are shown in Table 1. Of the sample, 36.7 % fulfilled the criteria for at least one lifetime disorder. The most common diagnoses were specific phobia and major depressive disorder (17.4 and 13.8 % respectively).

**Table 1:** Lifetime prevalence of DSM-IV mental disorders at baseline

Diagnosis	N	%
Major depressive disorder	300	13.8
Dysthymia	21	1.0
Generalized anxiety disorder	34	1.6
Social anxiety disorder	74	3.4
Specific phobia	378	17.4
Panic disorder	54	2.5
Agoraphobia	75	3.4
Alcohol use disorder	191	8.8
At least one of the above	1016	36.7

Having had any period of sick leave was common; during the entire follow-up period, 52.1 % of the sample had at least one period of sick leave exceeding 16 days. During an average year, 15.0 % of the sample was registered with sick leave. In total, the average proportion of workdays lost to sick leaves exceeding 16 days was 4.6 %. The occurrences of sick leave registered as granted for the different diagnostic categories are shown in Table 2.

**Table 2:** Proportion of lost workdays and individuals with sick leave granted for the different diagnostic categories

Officially registered sick leave diagnosis	Lost workdays	Individuals with sick leave in an average year	Individuals with sick leave during follow-up
All-cause	4.6 %	15.0 %	52.1 %
Mental	1.2 %	3.6 %	16.5 %
Somatic	3.0 %	10.8 %	41.6 %

Note: All-cause sick leave includes sick leaves with unknown diagnosis (6.9 % of sick days). Pregnancy related sick leave is not included in any of the variables

The associations between each mental disorder during the lifetime and later sick leave are shown in Table 3. When each disorder was analysed separately and adjusted for sex, age, and parental education, all eight mental disorders were associated with an increased rate of sick leave. The strongest association was seen with dysthymia (*RR* = 2.9), while more common disorders, such as specific phobia, showed a more moderate association with sick leave (*RR* = 1.7). The weakest association was seen for alcohol use disorder (*RR* = 1.5). Adjusting for all other mental disorders, only four of the disorders remained significantly associated with sick leave (major depressive disorder, dysthymia, social anxiety disorder and specific phobia). Ad-hoc analyses on specific phobia indicated that the increased rate of sick leave was not restricted to subjects who reported multiple phobias (*RR* = 1.6; 95 % CI 1.2 to 2.2; *p* = 0.003; *n* = 109), but was also found among subjects who had experienced only one specific phobia (*RR* = 1.8; 95 % CI 1.4 to 2.2; *p* > 0.001, *n* = 408). Two-way interactions between the three demography variables (sex, age, and parental education) and the eight mental disorders were tested. Out of 24 tests, none had a p-value below 0.01. The number of diagnoses was associated with the level of sick leave. For each additional diagnosis, subjects had 32 % more sick leave (*RR* = 1.3; 95 % CI 1.2 to 1.4, *p* < .001).

**Table 3:** Associations between eight lifetime mental disorders and sick leave

	Partially adjusted		Adjusted	
	RR	(95 % CI)	RR	(95 % CI)
All – cause sick leave				
Major depressive disorder	1.7	(1.4 – 2.1)	1.3	(1.1 – 1.7)
Dysthymia	2.9	(1.6 – 5.1)	2.1	(1.2 – 3.7)
Generalized anxiety disorder	2.2	(1.4 – 3.4)	1.2	(0.7 – 1.9)
Social anxiety disorder	2.2	(1.6 – 3.1)	1.5	(1.1 – 2.2)
Specific phobia	1.7	(1.4 – 2.1)	1.5	(1.2 – 1.9)
Panic disorder	1.6	(1.1 – 2.3)	0.9	(0.6 – 1.5)
Agoraphobia	1.7	(1.2 – 2.5)	1.3	(0.8 – 1.9)
Alcohol use disorder	1.5	(1.1 – 2.0)	1.2	(0.9 – 1.6)
Sick leave. mental disorders				
Major depressive disorder	3.4	(2.4 – 4.8)	2.4	(1.6 – 3.7)
Dysthymia	4.6	(1.7 – 12.3)	1.8	(0.6 – 5.2)
Generalized anxiety disorder	5.8	(3.0 – 11.2)	2.2	(1.1 – 4.6)
Social anxiety disorder	2.7	(1.5 – 4.7)	1.2	(0.7 – 2.3)
Specific phobia	1.7	(1.2 – 2.3)	1.2	(0.8 – 1.8)
Panic disorder	3.9	(2.1 – 7.3)	1.6	(0.6 – 4.3)
Agoraphobia	2.9	(1.7 – 5.1)	1.3	(0.5 – 3.5)
Alcohol use disorder	2.1	(1.2 – 3.7)	1.3	(0.7 – 2.3)
Sick leave. somatic disorders				
Major depressive disorder	1.2	(0.6 – 1.7)	1.0	(0.7 – 1.4)
Dysthymia	1.9	(0.7 – 4.8)	1.6	(0.6 – 4.2)
Generalized anxiety disorder	1.1	(0.5 – 2.1)	0.7	(0.3 – 1.5)
Social anxiety disorder	2.0	(1.3 – 3.3)	1.8	(1.1 – 2.9)
Specific phobia	2.0	(1.5 – 2.7)	1.9	(1.4 – 2.7)
Panic disorder	0.8	(0.4 – 1.4)	0.6	(0.3 – 1.2)
Agoraphobia	1.2	(0.7 – 2.0)	1.0	(0.6 – 1.8)
Alcohol use disorder	1.4	(0.9 – 2.2)	1.2	(0.8 – 2.0)

Note: Relative risk (RR) for being at sick leave on a given workday. All results are adjusted for sex, age, and parental education. Each disorder is adjusted for all the other disorders in the adjusted results. Confidence intervals (CI) with robust estimation. All-cause sick leave includes sick leave granted for mental and somatic disorders, as well as sick leaves where diagnosis was not available from the registries, but excludes pregnancy related sick leave. *N* = 2,178

All the eight mental disorders were associated with sick leave granted for mental disorders; generalized anxiety disorder and dysthymia most strongly. When all disorders were adjusted for each other and for demographics, major depressive disorder and generalized anxiety disorder had the strongest independent associations with sick leave granted for mental disorders. Social anxiety disorder and specific phobia had the strongest associations with sick leave granted for somatic disorders in both partially adjusted and adjusted analyses. The other disorders were not significantly associated with sick leave granted for somatic disorders, although the confidence intervals were relatively wide.

The PAFs of all-cause sick leave for each mental disorder are shown in Table 4. Specific phobia, major depressive disorder, and social anxiety disorder accounted for most sick leave, in that order.

**Table 4:** Population attributable fractions (PAFs) of all-cause sick leave by eight lifetime mental disorders

	PAF
Major depressive disorder	2.8 %
Dysthymia	0.9 %
Generalized anxiety disorder	0.4 %
Social anxiety disorder	1.5 %
Specific phobia	5.4 %
Panic disorder	-
Agoraphobia	0.7 %
Alcohol use disorder	0.8 %
Sum	12.5 %

## Discussion

In this study, we investigated the associations between eight common lifetime DSM-IV mental disorders and later sick leave granted for mental disorders, somatic disorders, and any disorder, in a population-based sample of young adult employees. All the examined disorders were prospectively associated with increased levels of sick leave, including sick leave granted for somatic disorders. Further, a large proportion of sick leave among young adult employees was attributable to lifetime occurrence of common anxiety disorders.

In line with previous studies, individuals who fulfilled criteria for present or previous mental disorders had higher levels of sick leave [1, 2, 7–10]. Mood and anxiety disorders predicted sick leave approximately to the same extent. This is inconsistent with results from a previous study, which showed that symptoms of anxiety, but not symptoms of depression, contributed to sick leave [7]. The discrepancy may be due to our use of lifetime diagnoses of mental disorders, rather than short-scale symptom measures. Unlike findings reported previously [4, 18–20], our results did not support the notion that mood disorders most strongly affect work-related impairment measures. Anxiety disorders could be more important for sick leave, compared to other impairment measures, due to the associated avoidance behaviour. In line with previous studies on comorbidity and impairment, sick leave also depended on the number of mental disorders [7, 18–20].

The high levels of sick leave among individuals with specific phobia may seem surprising, given that this disorder is often considered relatively mild [32]. Nevertheless, impairment is a diagnostic criterion also for specific phobia, and interestingly, two previous studies found the impairment among individuals with specific phobia to be on par with major depression and other internalizing disorders [17, 20]. Specific phobia can indicate a more general risk for psychopathology [33], and anxiety disorders can be an early sign of somatic illness [34]. It may therefore be time to acknowledge that specific phobia, although described as relatively circumscribed fears, can be associated with wider functional impairments. Unlike a previous study, we did not find that a higher number of specific phobia subtypes was associated with greater disability [35].

The relatively small association between alcohol use disorder and sick leave is in line with studies finding no or small associations between this disorder and work disability [17–21]. In our study, this may be due to the young age of the participants, because the detrimental health effects of alcohol develop over time [36]. In addition, short-term effects of alcohol are unlikely to be registered, as we only included spells of sick leaves exceeding 16 days. Alcohol use may, however, indirectly exert an effect on sick leave by increasing the risk for other mental disorders, in line with the partially adjusted results.

In our study, mental disorders correlated not only with sick leave granted for mental disorders, but also with sick leave granted for disorders in other diagnostic chapters. This finding is in accordance with previous studies of mental disorders and sick leave or disability pensions [1, 5, 6, 13, 37, 38]. Our results indicated that different mental disorders were unequally associated with sick leave officially granted for mental and somatic conditions. Mood and anxiety disorders may be divided into those characterized by distress (major depressive disorder, dysthymia, and generalized anxiety disorder) and those characterized by fear (panic disorder, agoraphobia, social anxiety disorder, and specific phobia) [39]. Our adjusted results indicated that disorders related to distress rather than fear had the strongest associations with sick leave explicitly granted for mental disorders. These disorders are characterised by fatigue, sleep disturbances, low energy levels, and difficulties concentrating, according to diagnostic criteria, and may therefore affect work capacity more immediately than disorders characterised by fear. In contrast to this, phobic anxiety disorders, i.e., social anxiety disorder or specific phobia, did not significantly contribute to sick leave granted for mental disorders, according to the adjusted results. Instead, individuals who experienced these disorders had higher rates of sick leave granted for somatic disorders. Social anxiety disorder is characterized by fear of social situations, whereas specific phobia is characterised by fear of specific objects or situations, such as heights, animals, or blood. These disorders may not justify sick leave per se, but are likely to have a more indirect association with work absence, for several reasons. First, mental disorders are frequently co-occurring with musculoskeletal and cardiovascular disorders [34, 40]. Thus, mental disorder may indicate a higher prevalence of somatic illnesses. Second, mental disorders can lead to poor coping skills or somatization and thereby impair an individual’s ability to work when having other disorders [10, 41]. Thus, co-existing mental disorders can influence whether individuals with somatic disorders go to work or not. Third, diagnostic practice could also influence the pattern of association [42]. Consider two patients who have back pain: One of them also has specific phobia and the other has generalized anxiety disorder. In both cases, the mental disorder can affect work functioning. As specific phobia is never the official reason for sick leave, the first patient will most likely get a musculoskeletal diagnosis. In the second case, both mental and somatic diagnoses are likely as registered reasons for sick leave. Also, general practitioners may not want to reinforce avoidance behaviour by granting sick leave for social or specific phobia, but may be willing to grant sick leave for a co-occurring somatic condition.

The PAFs were determined by RR and prevalence and indicated that specific phobia accounted for the highest proportion of sick leave, followed by major depressive disorder and social anxiety disorder. The PAFs reflect the proportion of sick leave preventable if a disorder is eradicated, given that the association is causal. However, the association between mental disorders and sick leave is not necessarily fully causal, but could be partially due to shared risk factors, or confounders, that influence both mental disorders and sick leave. In an Australian study with annual assessment of mental health and sick leave, results from regression analysis with correlated random effects suggested that a large part of the association between mental health and sick leave was confounded by unobserved differences between individuals [43]. In line with this, we have previously found that the association between mental health and sick leave granted for somatic disorders was confounded by familial factors, whereas the association with sick leave granted for mental disorders seemed to be causal [3]. Unmeasured confounders could lead to an overestimation of the proportion of sick leave preventable by treating mental disorder. At the same time, mental disorders are not independent constructs. Some risk factors are common to different mental disorders, and one disorder may develop as a response to another disorder, because of pathoplastic processes [44]. Fully adjusted results may therefore underestimate the effects of primary disorders. Although there are uncertainties associated with the PAFs, it is plausible that the association is partly causal, and the results at least identify risk groups. Healthcare personnel should therefore acknowledge the role co-occurring anxiety disorders might have in decreasing work capacity among individuals with somatic disorders. Specific phobia disorder is relatively easily treatable [45] and may be a target of intervention in itself. Specific phobia could also reflect neuroticism or a general tendency for psychopathology, in which case its presence could serve as a warning signal for other pathology. Employers, government, and insurers could nevertheless benefit from allocating more resources to individuals with specific phobia, trying to prevent sick leave within this group. Further research is needed to clarify the issue of causality.

The assessment of lifetime, rather than prevalent, mental disorders in the present study implies that the disorders were not necessarily present at the time of interview. A transient episode of a mental disorder is unlikely to affect future sick leave by itself. However, neuroticism, an underlying risk factor for mental disorders, is more stable [46], and a general and partially genetic risk for psychopathology can affect individuals throughout life [33]. Recurring episodes are also common [47]. Thus, individuals with a history of psychopathology are at risk, even if they have no current mental disorders. This being said, most mental disorders reported in the present study are likely to have been present during the last few years. Recent disorders are more likely to be remembered and reported, and up to two thirds of reported lifetime disorders were present during the past year [48], possibly because current mental health affects report of previous events [49].

The results are specific for sick leave and do not necessarily reflect the full occupational impairment associated with each mental disorders. Mental disorders increase the risk of being underemployed [50, 51], and one has to be employed to be eligible for sick leave. High employment rates among individuals with specific phobias may partly explain the relatively strong association between specific phobias and sick leave. Phobias are nevertheless related to a higher risk for developing other anxiety disorders during the follow-up time [23]. This serves as a reminder that occupational health services may need to prioritize differently from general health services or health services tailored for the unemployed.

### Limitations

The strengths of the present study include the use of structured diagnostic interviews, a prospective design, highly reliable registry data, and a sample recruited from the general population. Nevertheless, some limitations must be mentioned: First, the sample size was moderate and the number of cases was limited for some of the disorders. Second, fewer men than women participated in the study. High non-response rates are common among young men [52], and the overrepresentation of women may have biased the results. However, we found no significant interactions between sex and mental disorders on sick leave, indicating that mental disorders are associated with sick leave to the same degree among men and women. Moreover, the total rate of sick leave was 4.6 % and is comparable to the expected level in the Nordic countries [53]. The overrepresentation of women may also have led to inflated estimates of the prevalence of mood and anxiety disorders, whereas the prevalence of AUD could be underestimated. The total prevalence estimate of 36.7 % is nevertheless comparable to other studies [54]. Third, we did not have data on potentially important mediating or confounding variables. Future studies would benefit from including measures of somatic disorders, personality, and coping, as well as occupation and other work-related variables. Fourth, the observed associations do not necessarily reflect fully causal relationships. Although the sample consists of twin pairs, insufficient power prevents reliable adjustment for genetic and shared environmental confounders by running discordant twin analyses for specific diagnoses. This is because of the low number of discordant twins and the many possible combinations of disorders in a twin pair. Fifth, we only had data on long-term sick leave, lasting for at least 16 days. A previous meta-analysis has shown that “psychological problems” are more strongly associated with long-term than with short-term sick leave [9]. Sixth, generalizability may be limited by age of the respondents, culture, and welfare schemes.

## Conclusions

In conclusion, our study responds to previous limitations by using diagnostic interviews and registry based outcomes in a population-based sample. We have demonstrated that both mood and anxiety disorders constitute independent risk factors for all cause sick leave, whereas alcohol use disorders seemed to be of less importance in young adulthood. Major depressive disorder and generalized anxiety disorder were most strongly associated with sick leave granted for mental disorders, whereas specific phobia and social anxiety disorder primarily predicted sick leave granted for somatic conditions. Due to the high prevalence, a large part of all sick leave is related to specific phobia. The impairment associated with this common disorder may have been under-acknowledged, and its presence could serve as a warning signal of a more general risk of pathology or decrease work capacity among individuals with somatic disorders. This disorder has good treatment response, and may be overlooked as a target for interventions aimed at prevention of sick leave.

## Abbreviations

DSM, Diagnostic and Statistical Manual of Mental Disorders; ICD, International Classification of Diseases; ICPC-2, International Classification of Primary Care-2; PAF, Population attributable fractions; RR, Relative risk; WHO, World Health Organization
